# Re: Antibiotic prophylaxis prior to urodynamic study in patients with traumatic spinal cord injury. Is there an indication?

**DOI:** 10.1590/S1677-5538.IBJU.2019.0193

**Published:** 2019-09-02

**Authors:** Michael S Floyd, Rauf N. Khadr

**Affiliations:** 1 Department of Urology & Northwest Regional Spinal Cord Injury Unit Southport & Ormskirk NHS Foundation Trust, Town Lane, Kew, Southport, Merseyside, United Kingdom

*To the editor,*

We read with interest the recent paper by da Silva et al. examining effects of antibiotic prophylaxis and risk of urinary tract infection for spinal cord injured patients undergoing urodynamic studies. The authors describe a multi institutional study involving 661 patients who underwent urodynamic evaluation over 2 years ( 1 ). Three different antibiotic protocols are described in separate institutions and a cumulative infection rate of 3.18% was found. No differences between patient age or ASIA classification were found to have an association with the development of subsequent urinary tract infection. However, patients with injuries at T6 or above were at increased risk of developing urinary tract infection following urodynamic evaluation ( 1 ).

The authors are to be commended for conducting this study as there remains a paucity of literature regarding the topic with only 1 trial to date examining the topic ( 2 ).

The authors should acknowledge that the length of time between injury, first and subsequent urodynamic evaluation is not recorded and the rate of autonomic dysreflexia (if any) is not mentioned. It is stated that in the consideration of variables a numbers that several factors were included yet there is no baseline assessment of subjective symptoms based on patient questionnaires such as the neurogenic bladder symptom score ( 3 ). In the spinal cord injured patient videourodynamic assessment is the preferred method of urodynamic assessment.

Specific to our Spinal cord injury unit we routinely perform videourodynamic evaluation of spinal cord injured patients both as inpatients and outpatients and all undergo mandatory dipstick assessment prior to the procedure. If suggestive of infection the procedure is deferred but we do not prescribe antimicrobials pre investigation. Additionally we record bladder symptom scores at baseline with a validated questionnaire (SF Qualiveen) and repeat scores following definitive treatment to evaluate response ( 4 ).

Yours Sincerely,

Authors
